# Cerebral Sinovenous Thrombosis in Neonates: A Report of Three Cases

**DOI:** 10.7759/cureus.78833

**Published:** 2025-02-10

**Authors:** Olfa Asbik, Sahar Messaoudi, Mohammed Ech-Chebab, Chaymae Yechouti, Anass Ayyad, Rim Amrani

**Affiliations:** 1 Mother and Child Health Laboratory, Faculty of Medicine and Pharmacy, Mohammed First University, Oujda, MAR; 2 Department of Neonatology and Neonatal Resuscitation, Mohammed VI University Hospital, Oujda, MAR

**Keywords:** anticoagulation, cerebral sinovenous thrombosis, newborn, pediatric brain mri, perinatal stroke

## Abstract

The clinical aspects of neonatal cerebral sinovenous thrombosis (CSVT) are polymorphic in their onset and acute phase, making diagnosis challenging. The aim of our study is to shed light on this pathology through three clinical cases. This is a case series study of three cases of CSVT in newborns collected in the neonatology and neonatal intensive care unit at CHU Mohammed VI Oujda over a one-year period. The first patient was a male with a neonatal infection of indeterminate origin. He presented with convulsions, and a cerebral MRI revealed CSVT of the superior longitudinal sinus. The permeabilization of the venous sinus marked the course. The other two patients presented with CSVT, complicating bacterial meningitis. One of them, whose thrombosis had spread to multiple sinuses and was associated with ventriculitis, had a poor prognosis with thrombus extension and was scheduled for surgery. The second patient, whose thrombosis was confined to the superior longitudinal sinus, had a favorable outcome with complete repermeabilization of the sinus.

## Introduction

Cerebral sinovenous thrombosis (CSVT) is a very rare condition, with a rate of 0.7 per 100,000 children per year and a higher incidence in neonates (2.6 to 2.69 per 100,000 newborns per year) [1]. However, it may be underreported due to diagnostic complexity and clinical variability [1]. CSVT manifests as the occurrence of a thrombus within a cerebral venous structure, causing an obstruction to cerebral venous return and resulting in cerebral parenchymal damage of varying intensity, ranging from isolated intracranial hypertension to parenchymal ischemia or hemorrhage. In neonates, it is classified within the broader category of perinatal stroke and recurs less commonly than arterial ischemic stroke [1]. Clinically, CSVT can present with neurological signs such as seizures, lethargy, and focal neurological deficits, as well as nonspecific symptoms, making early diagnosis challenging [1]. The etiology of neonatal CSVT is multifactorial, typically involving genetic predispositions that interact with acquired risk factors such as perinatal infections, dehydration, and umbilical catheterization, and it can even occur as a complication of lumbar puncture [2]. In this series of case reports, we present three cases of neonatal CSVT, focusing on clinical presentations, diagnostic modalities, clinical interventions, and both short- and long-term prognoses observed in our clinical practice.

## Case presentation

The three cases of neonatal CSVT reported here were observed consecutively in our department between December 2020 and December 2021. They were included without specific selection, representing the most recent cases managed before the writing of this article.

Case 1

**Figure 1:**
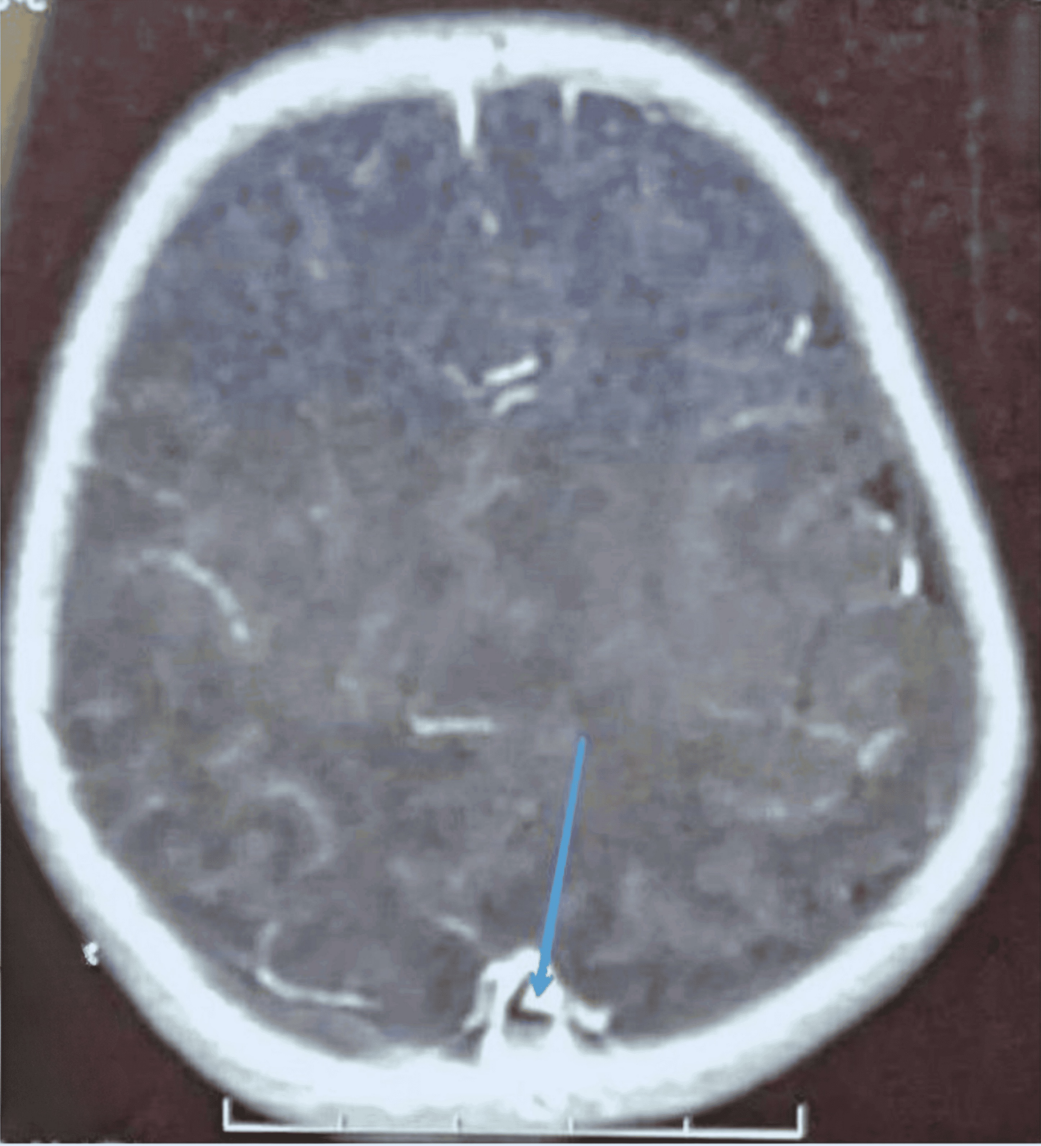
MRI of the patient on day 3 of life in T1 sequence with gadolinium injection, axial view, showing thrombosis in the superior sagittal sinus (blue arrow) MRI: magnetic resonance imaging

A three-day-old male baby, born at term (40 weeks gestation), was admitted to the neonatal intensive care unit following convulsive seizures. His medical history indicated signs of a probable infection, including colored amniotic fluid and fever during labor. A physical examination revealed weak primitive reflexes, hyporeactivity, mild hypotonia, and a bulging anterior fontanelle with a sunset gaze. Additional findings suggestive of a polymalformative syndrome included a heart murmur and various dysmorphic features: hypertelorism, low-set ears, brachydactyly, clinodactyly, right choanal atresia, and clubfoot. Systemic examinations were otherwise normal. Blood investigations revealed significant abnormalities, including slightly elevated C-reactive protein (CRP) and procalcitonin (PCT) levels (Table 1). However, lumbar puncture and urinalysis were negative for infection. Neonatal infection of unknown origin was diagnosed as the underlying condition. Radiological evaluation included a transfontanellar ultrasound with a complementary MRI, confirming neonatal CSVT (Figure 1). The neonate was treated with phenobarbital for seizure control and a 10-day course of a third-generation cephalosporin and ampicillin combination for the presumed infection. The infant showed significant improvement, with seizures resolving, skin color normalizing, and CRP levels returning to normal (Table 1). After 32 days, magnetic resonance angiography (MRA) follow-up demonstrated complete thrombosis resolution. Due to the presence of congenital abnormalities, an underlying genetic disorder was suspected. Genetic analysis confirmed partial monosomy of chromosome 13.

**Table 1:** Summary of observations of the three patients RPM: rupture of membranes, PC: perimeter cranien (head circumference), ETF: transfontanellar ultrasound, MRI: magnetic resonance imaging

	Patient 1	Patient 2	Patient 3
Sex	Male	Female	Male
Gestational age	40 weeks + 4days	39 weeks	34 weeks
Admission age	3 days	14 days	32 days
Reason for hospitalization	Convulsions	Convulsions + fever	Neonatal respiratory distress
Maternal history and delivery	Positive infectious history	Cesarean section	RPM > 18 hours
Temperature	36°C	39°C	37.5°C
Neurological examination	Hypotonic, hyporeactive, PC = 32.5 cm	Hypotonic, hyporeactive, PC = 42 cm	Hypotonic, hyporeactive, PC = 33 cm
Malformative assessment	Dysmorphic face, limb anomalies	Dysmorphic face, a lower incisor	Negative
ETF	Normal	Triventricular hydrocephalus	Normal
Cerebral MRI	Cerebral thrombophlebitis, Dandy-Walker malformation	Meningoencephalitis complicated by cerebral thrombophlebitis	Cerebral thrombophlebitis
Affected sinuses	Superior sagittal sinus	Superior sagittal sinus, lateral sinuses, torcular	Superior sagittal sinus
Final diagnosis	Neonatal infection of unknown focus, cerebral thrombophlebitis, monosomy 13	Neonatal meningitis complicated by cerebral venous thrombosis, ventriculitis	Neonatal respiratory and meningeal infection complicated by cerebral thrombophlebitis
Treatments received	Antibiotics + antiepileptic	Antibiotics + antiepileptic	Antibiotics
Outcome	Complete repermeabilization (day 28)	No repermeabilization	Complete repermeabilization (day 32)

Case 2

**Figure 2:**
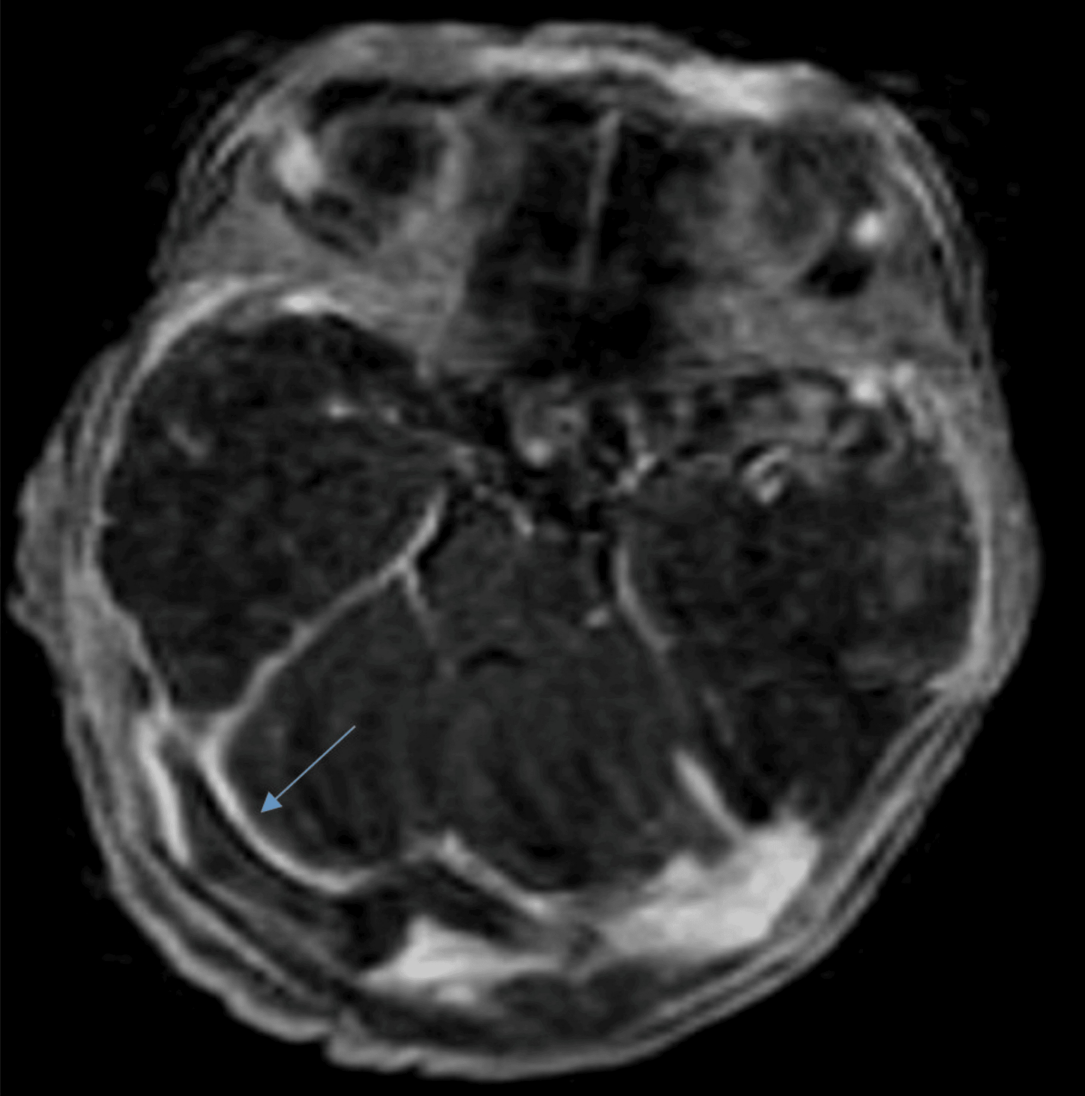
Brain MRI in axial T1 sequence with gadolinium injection showing thrombosis of the lateral sinuses (blue arrow) MRI: magnetic resonance imaging

**Figure 3:**
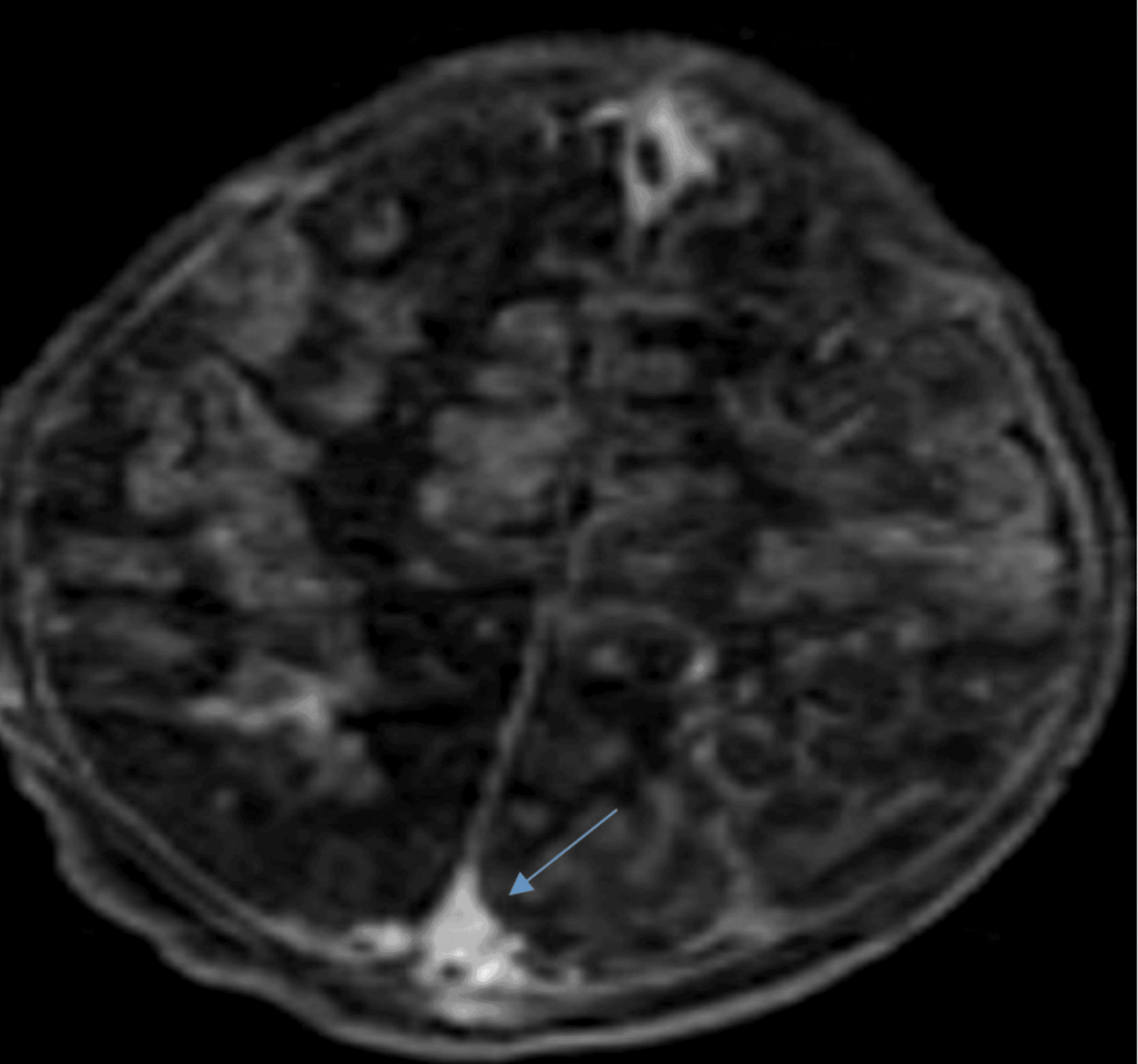
Brain MRI in axial T1 sequence with gadolinium injection showing thrombosis of the superior sagittal sinus (blue arrow) MRI: magnetic resonance imaging

A 14-day-old female newborn, born at term 39 weeks of gestation after a well-monitored pregnancy, was admitted to the neonatal intensive care unit due to seizures and fever. She had been delivered by cesarean section due to macrocephaly, with no history of infection. On admission, her vital signs were stable, with a temperature of 39°C. A neurological examination revealed a conscious newborn with a weak cry, hypotonia, hyporeactivity, intact sucking, and primitive reflexes. The anterior fontanel appeared normal. No major congenital malformations were detected; however, the baby presented with a dysmorphic facial appearance, including a lower incisor. Other systemic examinations were normal. Blood tests showed abnormalities, including elevated inflammatory markers such as CRP and PCT (Table 1). CSF analysis revealed turbidity, elevated white blood cell counts, low glucose levels, and high protein levels. Cultures grew *Pseudomonas aeruginosa*. Cerebral MRA revealed CSVT in the lateral sinuses, torcular, and superior sagittal sinus (Figures 2-3), with abnormal signals in multiple brain regions consistent with ischemia and meningoencephalitis complicated by thrombophlebitis. Triventricular hydrocephalus was also observed.

**Figure 4:**
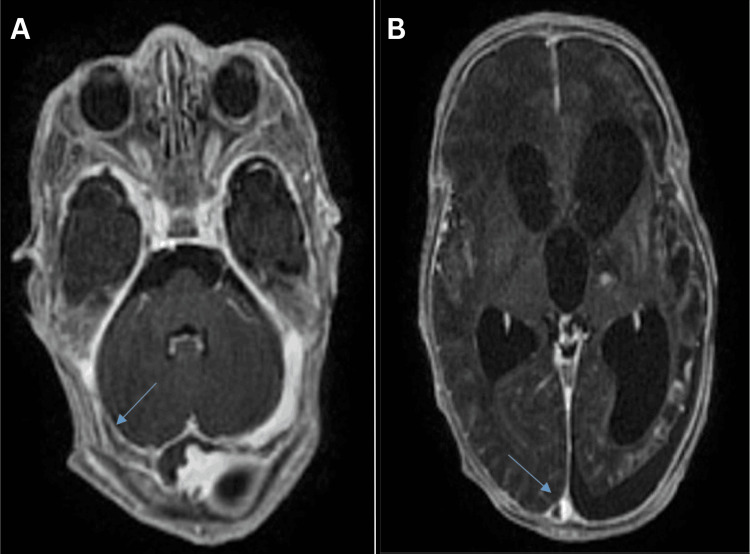
(A) Brain MRI in axial T1 sequence with gadolinium injection showing persistence of endoluminal material within the lateral sinuses (blue arrow). (B) Brain MRI in axial T1 sequence with gadolinium injection showing persistence of endoluminal material within the superior sagittal sinus (blue arrow). MRI: magnetic resonance imaging

The infant was diagnosed with meningoencephalitis, complicated by CSVT in multiple sinuses and secondary cerebral ischemia. She was treated with analgesics and phenobarbital for seizures, and antibiotics were adjusted according to the antibiogram. After 10 days of hospitalization, the baby remained hypotonic and hyporeactive; however, inflammatory markers, including CRP and PCT, had significantly decreased (Table 1). A follow-up cerebral MRA confirmed persistent thrombosis in the lateral sinuses, torcular, and superior sagittal sinus, along with hemorrhagic infarcts. Triventricular hydrocephalus remained without signs of transependymal resorption. Additionally, two new bilateral capsulo-lenticular ring-enhancing lesions were observed, likely representing suppurative foci (Figure 4). Ceftriaxone treatment was extended due to ventriculitis, and the decision was made to transfer the patient to the neurosurgery department for further management, including the placement of a ventriculoperitoneal shunt once the CSF was sterilized.

Case 3

A 32-day-old male infant, born at 34 weeks gestation as a late preterm baby, was admitted for neonatal respiratory distress. The pregnancy had been closely monitored, and delivery occurred vaginally. The infant's medical history included premature rupture of membranes lasting over 18 hours and the presence of discolored amniotic fluid. Upon admission, clinical assessment revealed a respiratory rate of 60 breaths per minute, a heart rate of 118 beats per minute, and a temperature of 37.5°C. A pleuro-pulmonary examination noted cyanosis with a Silverman score 5/10, normal auscultation, and stable oxygen saturation. Neurologically, the infant was conscious but exhibited a weak cry, reduced reactivity, hypotonia, and no motor deficits, with intact sucking, swallowing, and primitive reflexes. No congenital malformations were identified, and other systemic examinations were unremarkable.

Laboratory results showed elevated inflammatory markers, including CRP and PCT levels (Table 1). Cerebrospinal fluid analysis revealed turbid fluid with increased white blood cells (95% lymphocytes), red blood cells, normal glucose levels, and elevated protein content. Cultures confirmed the presence of *Streptococcus* spp.

**Figure 5:**
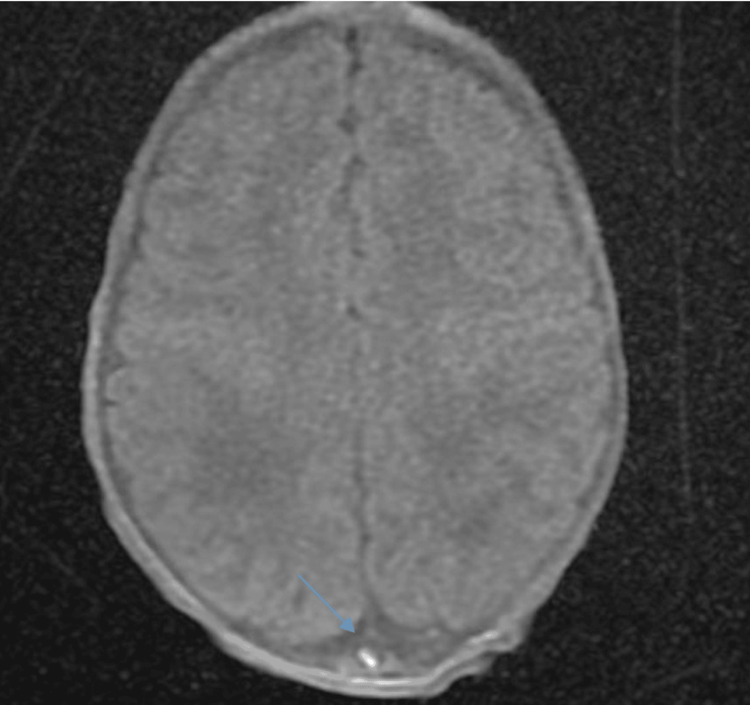
Brain MRI in axial T1 sequence without gadolinium injection showing a hyperintensity in the lower part of the superior sagittal sinus (blue arrow) MRI: magnetic resonance imaging

**Figure 6:**
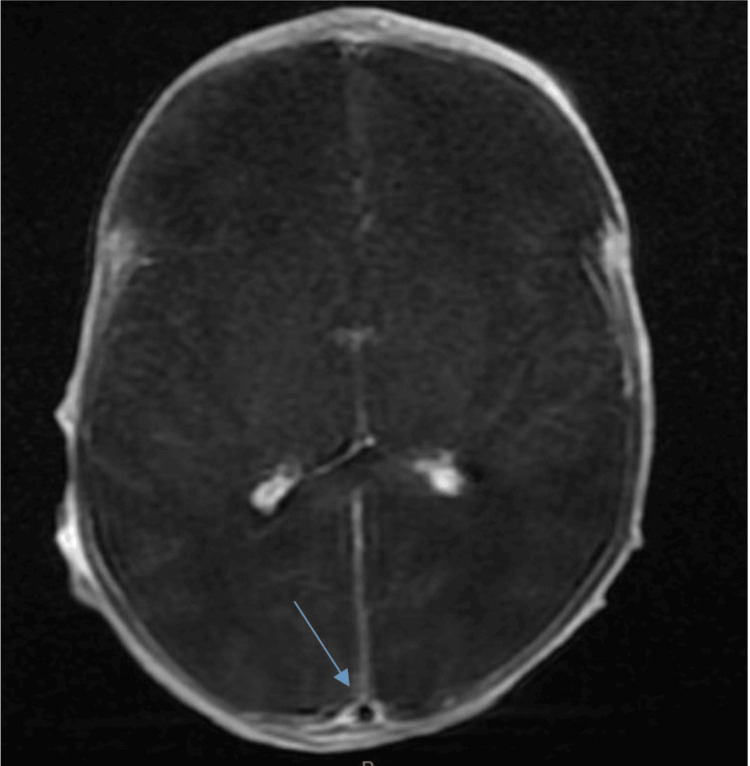
Brain MRI in axial T1 sequence with gadolinium injection showing the empty delta sign (blue arrow) MRI: magnetic resonance imaging

Imaging studies included a chest X-ray, which identified an infection in the right apical region, a normal cranial ultrasound, and a cerebral MRA performed on day 5 of hospitalization. This MRA, following the onset of generalized convulsive seizures, showed CSVT of the superior sagittal sinus, along with meningeal and intraventricular hemorrhage, but no signs of ischemia (Figures 5-6).

The initial treatment focused on stabilization with a heated table, monitoring, and oxygen therapy. The infant received a 21-day course of ceftriaxone and ampicillin. After 32 days of hospitalization, the patient showed significant clinical improvement, becoming tonic, reactive, and afebrile. Follow-up tests (Table 1) and imaging showed marked recovery. The chest X-ray showed improvement, and the cerebral MRA revealed no further signs of CSVT, though signs of meningeal and intraventricular hemorrhage remained stable.

Table 3 shows the laboratory parameters and biological results comparing day 1 and day 10 values for the three patients against normal and reference ranges.

**Table 2:** Biological results CRP: C-reactive protein, PCT: procalcitonin

Lab parameters (units)	Patient's normal values						Reference range
	Patient 1		Patient 2		Patient 3		
	Day 1	Day 10	Day 1	Day 10	Day 1	Day 10	
CRP	22.10 mg/l	2.53 mg/L	253 mg/l	6.6 mg/l	10 mg/l	5 mg/l	0.00-5.00 mg/l
PCT	1.40 ng/ml	0.03 ng/ml	13.29 ng/ml	0.19 ng/ml	1.37ng/ml	0.09 ng/ml	<0.1 ng/ml

## Discussion

Neonatal CSVT is a rare but serious condition, with an underestimated incidence ranging from 12 to 47/100,000 term neonates per year. However, it has a mortality rate of 50% of all pediatric CSVT cases, making this condition a significant cause of morbidity and mortality [1,3]. This underestimation can often be attributed to insidious, nonspecific clinical signs and variability in diagnostic and screening practices. Early diagnosis and prompt management are crucial, given the impact of this condition on the long-term neurological development of affected newborns.

The hemostatic system of newborns is characterized by decreased platelet reactivity and lower levels of several coagulation factors, suggesting a hemorrhagic phenotype. However, these findings are delicately balanced by other factors in neonatal blood that promote coagulation, such as increased hematocrit, mean corpuscular volume, von Willebrand factor, and low levels of natural anticoagulants [4]. Factors predisposing to the development of cerebral venous thrombosis (CVT) in newborns can be of maternal or neonatal origin. In our series, we identified premature rupture of membranes, prematurity, and meningitis as the main factors associated with the occurrence of CVT.

In newborns, CVT distribution appears to follow that of adults and older children, with slightly higher involvement of the superficial venous system of the brain, likely due to its vulnerability to mechanical forces during delivery. Molding and overlapping cranial sutures during complicated deliveries can lead to compression or damage of the underlying venous sinuses, thereby promoting thrombosis [2].

The clinical manifestations of CVT in newborns are highly variable and depend on several factors, such as the location and extent of the thrombosis, the patient’s age, the nature of the underlying condition, and the speed of thrombosis formation. Seizures are the most common clinical manifestation of neonatal CVT, occurring in up to 70% of cases in some studies [5]. These seizures may be generalized or focal and typically appear after a median interval of 1.5 days.

MRI, with its combination of sequences and high sensitivity to the magnetic susceptibility of blood degradation products, is the examination of choice for diagnosing CVT and associated lesions. CVT on MRI may be suspected by direct visualization of the thrombus, the absence of venous filling, and imaging of the consequences of venous obstruction at the tissue level (venous infarction, edema, hemorrhagic transformation, intracranial hypertension, and hydrocephalus) and at the vascular level (dilated veins) [6].

Thrombi can also be directly observed on conventional MRI sequences. Because the evolution of a thrombus on MRI is dynamic, changes in the signal intensity of the thrombus over time are similar to that of a hematoma. As the thrombus ages, oxyhemoglobin is converted to deoxyhemoglobin and methemoglobin, leading to changes in signal characteristics on the T1 and T2 sequences [7].

A definitive diagnosis is established when magnetic resonance venography demonstrates a clear absence of flow in a sinus at thresholds of 300 and 150 mm/s, combined with signal abnormalities on T1- and T2-weighted images suggesting thrombosis [8].

The use of anticoagulant therapy for neonatal CVT remains controversial due to several challenges, including a lack of safety data, the risk of spontaneous intracranial hemorrhages, uncertainty about the appropriate duration of treatment, and the absence of long-term randomized controlled trials (RCTs) in neonates. According to the American College of Chest Physicians 2012 guideline, anticoagulants are recommended in newborns and children in CVT without significant hemorrhage. Initially, UFH or LMWH is used, followed by LMWH or oral anticoagulants for at least six weeks in newborns and three months in older patients [9]. If there is complete recanalization or symptoms persist after three months, anticoagulation should continue for an additional three months. The presence of minor bleeding does not contraindicate the use of anticoagulants, while significant intracranial hemorrhage is considered a relative contraindication. Radiological surveillance should be performed for five to seven days, and anticoagulation should be initiated in case of thrombus propagation.

In the context of current recommendations and the absence of RCTs, the decision to initiate treatment is made on a case-by-case basis, considering factors such as the location and extent of the thrombus, the presence of intracranial hemorrhage, the reversibility of risk factors, and the ability to monitor anticoagulant therapy. In all three cases, no anticoagulant treatment was administered. Two cases showed favorable outcomes, while in the third case, anticoagulation was not initiated due to the presence of hemorrhage.

The enduring consequences for children who survive CSVT remain heterogeneous [10]. The variability in outcomes is largely due to the lack of standardized protocols for investigating neurological sequelae. The rates of neurological sequelae reported range from 40% to 80%, with the most common complications being epilepsy, cognitive deficits, and motor disorders [10]. These include a wide range of complications, from cerebral palsy to language disorders, which are of paramount importance in the quality of life of patients and the long-term burden of care. In our cases, we noted gross motor impairment and psychomotor developmental delay as complications, which further emphasized the need for an individualized follow-up and management strategy.

At each level of care, prevention should be considered, targeting primary, secondary, and tertiary prevention. Strategies for primary prevention should focus on decreasing risk factors during the perinatal period, such as maternal diabetes and hypertension, and preventing obstetric interventions that increase the risk of CSVT. Additionally, transfontanellar Doppler ultrasound should be implemented for screening. Secondary prevention refers to neuroprotection and the specialized care of the affected newborn. In contrast, tertiary prevention involves informing the parents about the need for early rehabilitation and follow-up to minimize neurological sequelae to the lowest possible level.

## Conclusions

Neonatal CSVT presents a challenge for early diagnosis and treatment due to its complexity and potential consequences on neurological development. Prompt diagnosis and appropriate treatment as soon as possible are key factors for better outcomes. The implementation of standardized protocols for both diagnosis and follow-up, along with the performance of multicenter studies, are steps that need to be considered to optimize the management of the condition. Additionally, raising awareness among healthcare professionals and parents, along with a multidisciplinary approach, is critically important in ensuring the best long-term prognosis for these affected children.
